# Transcriptional Differences in Identical Twins With Different Reproductive Capacities: A Case Report

**DOI:** 10.7759/cureus.40847

**Published:** 2023-06-23

**Authors:** Katherine Campbell, Alexandra Dullea, Christian Ramsoomair, Kyle Schuppe, Armin Ghomeshi, Kajal Khodamoradi, Himanshu Arora, Carolina Jorgez, Ranjith Ramasamy

**Affiliations:** 1 Urology, University of Miami Desai Sethi Urology Institute, Miami, USA; 2 Medicine, Washington State University Elson S. Floyd College of Medicine, Spokane, USA; 3 Psychiatry, Florida International University, Herbert Wertheim College of Medicine, Miami, USA; 4 Urology, University of Miami Miller School of Medicine, Miami, USA; 5 Urology, University of Miami, Miami, USA; 6 Urology, Baylor College of Medicine, Houston, USA

**Keywords:** case report, hox, e2f1, azoospermia, genetics, infertility

## Abstract

Disorders of sperm production can be classified quantitatively as oligospermia (low sperm count) or azoospermia (no sperm during ejaculation). Numerous genes have been implicated in spermatogenesis. We describe a case of two identical twins who presented with different reproductive capabilities. One brother was infertile due to azoospermia, and the other, although oligospermic, previously naturally fathered a child. They were found to have differential gene expression based on RNA sequencing analysis. In the man with azoospermia, we found elevated *E2F1 *and *HOXB9 *gene expressions when compared with his brother, suggesting that the increased RNA expression of these genes could influence sperm production.

## Introduction

Male factor infertility has been found to be solely responsible for 20-30% of infertile couples and contributes to 50% of cases overall [1]. Qualitative and quantitative alterations in sperm parameters are possible, with quantitative disturbances classified based on severity. Azoospermia (no sperm present during ejaculation) has been identified in about 15% of infertile men and can be classified as obstructive azoospermia (OA) and nonobstructive azoospermia (NOA) [2].

More than 2,000 genes are involved in spermatogenesis; therefore, the pathophysiology underlying disorders of spermatogenesis represents a highly complex interplay between genetic alterations and environmental aberrations [3]. Mutations in numerous genes have been implicated in the development of various abnormalities in sperm development [4,5]. Based on prior animal studies, numerous genes have been identified that play a role in spermatogenesis; *E2F1* and *HOXB9* are thought to regulate the G1/S transition and gene transcription, respectively [6,7].

In addition to these genetic causes, the epigenetic control of gene expression, often resulting from environmental or lifestyle factors, also plays a role in the development of male infertility, but it remains poorly understood [8]. In this study, we assessed differences in the RNA expression between two genetically identical individuals with different reproductive phenotypes. We examined the transcriptome in a unique case of identical twins, one with oligospermia and the other with NOA. Here, we present the first report of a novel finding of alteration in gene expression between genetically identical siblings. This work, previously presented at 78th Scientific Congress of the American Society for Reproductive Medicine, on October 25, 2022, presents a unique case description of the post-transcriptional modification of gene expression leading to phenotypic changes.

## Case presentation

Patient selection

A 38-year-old Black male was identified as having NOA (testis volume of 10cc bilaterally and follicle stimulating hormone (FSH) of 14 IU/mL) during a routine male fertility evaluation. His twin brother was found to be oligospermic but had previously naturally fathered a child. Both patients denied personal history of organic cause of male infertility (testosterone use, infections, or testicular trauma). Both patients gave their consent to participate in the study. Institutional review board (IRB) approval (#20150740) was obtained from the University of Miami, Florida, United States.

RNA preparation

Peripheral whole blood samples were collected. Total RNA was isolated using the TRIzol method. Next, reverse transcription to complementary DNA was induced using High-Capacity cDNA Reverse Transcription Kits (Applied Biosystems, USA) according to the manufacturer’s protocol.

RNA sequencing

RNA sequencing was completed at the John P Hussmann Institute for Human Genomics at the University of Miami. FastQ files were put through the FastQC program (Babraham Institute, UK) to check for quality. The adapter trimming software Trimgalore (Babraham Institute, UK), which uses the cutadaptpython package, was used for trimming. Reads were aligned against the human genome assembly GRCh37 (hg19) genome with the STAR RNAseq aligner and quantified against the GENCODE v19 database. Post-alignment quality control was performed with Picard tools (Broad Institute, USA) to check for ribosomal contented transcriptome alignment percentages. After alignment, differential expression comparisons were made with the DESeq2 R package (Bioconductor Project, USA). Due to the lack of replicates, the results from the comparison included nominal p-value significance (>0.05) instead of false discovery rate (FDR)-corrected p-values, as well as normalized expression values. The normalized expression values were then put through a custom pipeline to determine patterns. First, minimum, maximum, and mean values were calculated from the four separate samples for each gene. Then, a standard deviation was calculated across the samples.

RNA analysis

A range of concentration expression values was formed using a maximum value of the baseline +0.75X standard deviation and a minimum value of the baseline -0.75X standard deviation. Different standard deviations were tested to check specificity and sensitivity levels, and 0.75X was determined to give the most viable results. The samples were lined up according to the concentration from zero to two. Comparisons to the baseline concentration classifications were calculated based on the comparison of the expression value to the calculated range. Those that were greater than the maximum range value were determined to be an upward change of expression, and those that were less than the minimum range value were determined to be a downregulation expression change in a given sample. Based on these patterns of change, we calculated the patterns that were of most clinical interest. Ingenuity Pathway Analysis (QIAGEN, USA) was used to enrich the selected markers from each section with respect to their involvement in molecular signaling networks.

RNA Analysis Results

Among the RNA sequencing results, we focused on protein coding genes with a fold change of greater than 10 times, resulting in 65 genes with 10-fold increase in expression and 132 with 10-fold decrease in expression (see Appendix). The resulting genes were subjected to enrichment using Ingenuity Pathway Analysis to identify the signaling pathways with which these candidates could be involved in. Ingenuity Pathway Analysis was utilized because it provides a robust platform for accurate gene expression analysis with a high degree of reproducibility. Importantly, the highlighted genes are involved in cell cycle regulation, which have previously been implicated in spermatogenesis. After comparing the RNAs of the brothers, we identified two genes differentially expressed in the NOA brother when compared to the oligospermic one. *E2F1* expression was increased by 18-fold and *HOXB9* expression by 11-fold in the NOA brother.

## Discussion

We identified two monozygotic twins with defects in sperm production, one of them lacking sperm production. To understand the biological difference underlying their phenotypes, we focused on determining differences in gene expression, and we identified differential RNA expression in these genetically identical patients. We found increased of *E2F1* and *HOXB9* expressions in the azoospermic subject when compared to his oligospermic brother, which could represent post-transcriptional modification or epigenetic changes. *E2F1* and *HOXB9* are genes that play a role in the regulation of the cell cycle. *E2F1* is a transcription factor that regulates heat shock protein 70 to control the G1/S checkpoint and assists with DNA repair, while *HOXB9* regulates gene transcription through binding at a promoter region [6,7].

*HOX* genes are commonly associated with embryonic development and axonal positioning, as well as cell growth and regulation into adulthood [9,10]. *HOXB9* is highly expressed in apoptotic cell lines and those with decreased proliferation and migration [11]. *HOXB9* is expressed in T1 prospermatogonia-like cells [5]. Recently, *HOXB9* was identified as a candidate gene responsible for male infertility in silico using cytogenetic data from cultured peripheral blood lymphocytes of infertile patients [12]. Based on these findings, the increase in *HOXB9* expression in our NOA subject suggests that *HOX* activation may play a role in his defectivespermatogenesis.

*E2F1* is a transcription factor regulator of spermatogenesis. *E2F1* knockout mice showed decreased spermatogenesis that worsened through their lifetime culminating in azoospermia, testicular atrophy, and infertility [13,14]. Similarly, in mice, when transgenic overexpression of *E2F1* was induced, testicular apoptosis was seen, resulting in azoospermia and infertility [15]. More importantly, microduplication and microdeletions of *E2F1* have been linked to human cases of NOA in up to 7% of patients [16]. Rocca et al. confirmed these findings by reporting an association between NOA and *E2F1* expression and hypothesizing that the copy number variants of *E2F1* increase susceptibility to heat stress, thus, potentially, contributing to impairment in spermatogenesis [17]. Due to the impacts of *E2F1* on germ cell survival and spermatogenesis, our finding of *E2F1* upregulation in the azoospermic subject is of clinical importance.

Our study is not without limitations. Importantly, as a case series with two participants, the sample size is very small. With this RNA analysis, we are unable to assess the modification of the expression of these genes or the mechanisms behind the RNA expression changes. While Ingenuity Pathway Analysis provides an extensive, curated database of gene interactions and molecular pathways to aid in the interpretation of the biological context of our dataset, a significant quantitative difference in gene expression (as is seen in our study) is not solely indicative of pathology. This is particularly true when considering normal alleles, even when heterozygous, as differences in expression can occur naturally without signifying disease. While the differences exhibited in our study are notable and warrant further investigation, the changes in expression, in isolation, do not necessarily constitute proof of pathology. For example, these expression variants could be a part of a compensatory mechanism or an unrelated physiological process. Further functional studies are needed to definitively attribute these gene expression changes to the condition under investigation.

Despite these limitations, this study poses a unique opportunity to analyze the epigenetics of two genetically identical but phenotypically different men. These results contribute to the body of work characterizing the genes involved in sperm development. The identification of genes and their post-transcriptional modifications that play a role in spermatogenesis, such as *HOXB9* and *E2F1*, provides further insights into the molecular mechanisms underlying sperm development. This case suggests that while genetic variations have been implicated in abnormalities of sperm development and function, epigenetic control of gene expression (which may be influenced by environmental or lifestyle factors) also plays a role in male fertility. Gene therapy targeting these specific genes may potentially have future implications on the management of infertile men. We hope that works at our institution and others can further elucidate the genes and mechanisms by which genetic variants influence sperm phenotypes.

## Conclusions

This work presents a unique opportunity to assess differences in RNA expression in identical twins. We identified differences in *HOXB9* and *E2F1* RNA expression in identical twins with differential reproductive capacities, suggesting that these genes may impact spermatogenesis. Our results recapitulated the results of prior studies, indicating that increased expression in *E2F1* cause azoospermia and infertility. Understanding the epigenetic basis of infertility is an important step in developing new treatments and interventions.

## Appendices

**Table 1:** Genes showing a decrease of more than 10 folds

Symbol	Type	Name	Location	202110653-06_CPM	202110654-06_CPM	Fold difference
ENSG00000134825.16	protein_coding	"TMEM258"	chr11:61768501-61792802	21.8561745	2.1644644	-10.097729
ENSG00000156587.16	protein_coding	"UBE2L6"	chr11:57551656-57568284	21.8561745	2.1644644	-10.097729
ENSG00000171298.13	protein_coding	"GAA"	chr17:80101556-80119881	21.8561745	2.1644644	-10.097729
ENSG00000218891.5	protein_coding	"ZNF579"	chr19:55576774-55580848	21.8561745	2.1644644	-10.097729
ENSG00000102996.5	protein_coding	"MMP15"	chr16:58025754-58046901	7.28539151	0.72148813	-10.097729
ENSG00000112149.10	protein_coding	"CD83"	chr6:14117256-14136918	7.28539151	0.72148813	-10.097729
ENSG00000120129.6	protein_coding	"DUSP1"	chr5:172768096-172771195	7.28539151	0.72148813	-10.097729
ENSG00000121900.19	protein_coding	"TMEM54"	chr1:32894594-32901438	7.28539151	0.72148813	-10.097729
ENSG00000123159.16	protein_coding	"GIPC1"	chr19:14477760-14496149	14.570783	1.44297627	-10.097729
ENSG00000130810.20	protein_coding	"PPAN"	chr19:10106362-10112012	7.28539151	0.72148813	-10.097729
ENSG00000137168.8	protein_coding	"PPIL1"	chr6:36854827-36874803	7.28539151	0.72148813	-10.097729
ENSG00000143816.8	protein_coding	"WNT9A"	chr1:227918656-227947932	7.28539151	0.72148813	-10.097729
ENSG00000145708.11	protein_coding	"CRHBP"	chr5:76953045-76981158	14.570783	1.44297627	-10.097729
ENSG00000153989.8	protein_coding	"NUS1"	chr6:117675469-117710727	7.28539151	0.72148813	-10.097729
ENSG00000161243.9	protein_coding	"FBXO27"	chr19:38990714-39032785	7.28539151	0.72148813	-10.097729
ENSG00000162729.14	protein_coding	"IGSF8"	chr1:160091340-160098943	7.28539151	0.72148813	-10.097729
ENSG00000164761.9	protein_coding	"TNFRSF11B"	chr8:118923557-118951885	14.570783	1.44297627	-10.097729
ENSG00000169740.14	protein_coding	"ZNF32"	chr10:43643860-43648881	7.28539151	0.72148813	-10.097729
ENSG00000170270.5	protein_coding	"GON7"	chr14:93202894-93207065	7.28539151	0.72148813	-10.097729
ENSG00000171401.15	protein_coding	"KRT13"	chr17:41500981-41505705	7.28539151	0.72148813	-10.097729
ENSG00000185686.18	protein_coding	"PRAME"	chr22:22547701-22559361	7.28539151	0.72148813	-10.097729
ENSG00000204936.10	protein_coding	"CD177"	chr19:43353686-43363172	7.28539151	0.72148813	-10.097729
ENSG00000260001.6	protein_coding	"TGFBR3L"	chr19:7916145-7919097	7.28539151	0.72148813	-10.097729
ENSG00000189043.10	protein_coding	"NDUFA4"	chr7:10931943-10940153	29.8038744	2.88595254	-10.327223
ENSG00000110888.17	protein_coding	"CAPRIN2"	chr12:30709552-30754951	30.4661827	2.88595254	-10.556716
ENSG00000162873.15	protein_coding	"KLHDC8A"	chr1:205336061-205357090	15.2330913	1.44297627	-10.556716
ENSG00000213420.8	protein_coding	"GPC2"	chr7:100169606-100177381	15.2330913	1.44297627	-10.556716
ENSG00000162704.16	protein_coding	"ARPC5"	chr1:183620846-183635783	38.4138825	3.60744067	-10.648514
ENSG00000152229.18	protein_coding	"PSTPIP2"	chr18:45983536-46072272	23.8430995	2.1644644	-11.015704
ENSG00000176083.18	protein_coding	"ZNF683"	chr1:26361634-26374522	23.8430995	2.1644644	-11.015704
ENSG00000101463.6	protein_coding	"SYNDIG1"	chr20:24469629-24666616	7.94769983	0.72148813	-11.015704
ENSG00000109103.12	protein_coding	"UNC119"	chr17:28546708-28552631	7.94769983	0.72148813	-11.015704
ENSG00000124875.10	protein_coding	"CXCL6"	chr4:73836640-73849064	7.94769983	0.72148813	-11.015704
ENSG00000132744.8	protein_coding	"ACY3"	chr11:67642555-67650730	7.94769983	0.72148813	-11.015704
ENSG00000136487.18	protein_coding	"GH2"	chr17:63880215-63881944	7.94769983	0.72148813	-11.015704
ENSG00000147121.16	protein_coding	"KRBOX4"	chrX:46447292-46497422	7.94769983	0.72148813	-11.015704
ENSG00000162222.14	protein_coding	"TTC9C"	chr11:62728069-62740293	7.94769983	0.72148813	-11.015704
ENSG00000170689.10	protein_coding	"HOXB9"	chr17:48621156-48626358	7.94769983	0.72148813	-11.015704
ENSG00000171135.15	protein_coding	"JAGN1"	chr3:9890574-9894349	7.94769983	0.72148813	-11.015704
ENSG00000172782.12	protein_coding	"FADS6"	chr17:74877302-74893781	7.94769983	0.72148813	-11.015704
ENSG00000188816.3	protein_coding	"HMX2"	chr10:123148122-123150672	7.94769983	0.72148813	-11.015704
ENSG00000265681.7	protein_coding	"RPL17"	chr18:49488453-49492523	7.94769983	0.72148813	-11.015704
ENSG00000167778.9	protein_coding	"SPRYD3"	chr12:53064316-53079404	15.8953997	1.44297627	-11.015704
ENSG00000173480.11	protein_coding	"ZNF417"	chr19:57900296-57916610	15.8953997	1.44297627	-11.015704
ENSG00000214706.10	protein_coding	"IFRD2"	chr3:50287732-50292918	15.8953997	1.44297627	-11.015704
ENSG00000279000.3	protein_coding	"OR10A6"	chr11:7924592-7931268	15.8953997	1.44297627	-11.015704
ENSG00000169679.15	protein_coding	"BUB1"	chr2:110637528-110678063	24.5054078	2.1644644	-11.321696
ENSG00000028137.19	protein_coding	"TNFRSF1B"	chr1:12166991-12209228	16.557708	1.44297627	-11.474692
ENSG00000131398.15	protein_coding	"KCNC3"	chr19:50311937-50333515	16.557708	1.44297627	-11.474692
ENSG00000132702.13	protein_coding	"HAPLN2"	chr1:156619331-156625725	16.557708	1.44297627	-11.474692
ENSG00000251664.5	protein_coding	"PCDHA12"	chr5:140875302-141012347	16.557708	1.44297627	-11.474692
ENSG00000136451.9	protein_coding	"VEZF1"	chr17:57971547-57988259	25.1677161	2.1644644	-11.627688
ENSG00000115657.14	protein_coding	"ABCB6"	chr2:219209772-219218994	8.61000815	0.72148813	-11.933679
ENSG00000124900.12	protein_coding	"TRIM51"	chr11:55883297-55891810	8.61000815	0.72148813	-11.933679
ENSG00000128645.15	protein_coding	"HOXD1"	chr2:176188668-176190907	8.61000815	0.72148813	-11.933679
ENSG00000138495.6	protein_coding	"COX17"	chr3:119654513-119677454	8.61000815	0.72148813	-11.933679
ENSG00000157368.10	protein_coding	"IL34"	chr16:70579895-70660682	8.61000815	0.72148813	-11.933679
ENSG00000180964.17	protein_coding	"TCEAL8"	chrX:103252995-103255192	8.61000815	0.72148813	-11.933679
ENSG00000183386.10	protein_coding	"FHL3"	chr1:37996770-38005606	8.61000815	0.72148813	-11.933679
ENSG00000244617.2	protein_coding	"ASPRV1"	chr2:69960089-69962265	8.61000815	0.72148813	-11.933679
ENSG00000156976.17	protein_coding	"EIF4A2"	chr3:186783205-186789897	17.2200163	1.44297627	-11.933679
ENSG00000261272.1	protein_coding	"MUC22"	chr6:31010474-31035402	17.2200163	1.44297627	-11.933679
ENSG00000003393.15	protein_coding	"ALS2"	chr2:201700267-201780956	35.7646493	2.88595254	-12.392667
ENSG00000070423.18	protein_coding	"RNF126"	chr19:647526-663233	17.8823246	1.44297627	-12.392667
ENSG00000135916.16	protein_coding	"ITM2C"	chr2:230864639-230879248	17.8823246	1.44297627	-12.392667
ENSG00000164112.13	protein_coding	"TMEM155"	chr4:121758930-121765427	17.8823246	1.44297627	-12.392667
ENSG00000111783.13	protein_coding	"RFX4"	chr12:106583004-106762803	36.4269576	2.88595254	-12.622161
ENSG00000116521.11	protein_coding	"SCAMP3"	chr1:155255979-155262430	9.27231647	0.72148813	-12.851655
ENSG00000125998.8	protein_coding	"FAM83C"	chr20:35285731-35292425	9.27231647	0.72148813	-12.851655
ENSG00000146232.17	protein_coding	"NFKBIE"	chr6:44258166-44265788	9.27231647	0.72148813	-12.851655
ENSG00000156206.14	protein_coding	"CFAP161"	chr15:81007033-81149179	9.27231647	0.72148813	-12.851655
ENSG00000106789.13	protein_coding	"CORO2A"	chr9:98120975-98192637	18.5446329	1.44297627	-12.851655
ENSG00000160256.13	protein_coding	"FAM207A"	chr21:44940012-44976989	18.5446329	1.44297627	-12.851655
ENSG00000196152.10	protein_coding	"ZNF79"	chr9:127424374-127445372	18.5446329	1.44297627	-12.851655
ENSG00000117425.14	protein_coding	"PTCH2"	chr1:44819844-44843253	19.2069413	1.44297627	-13.310643
ENSG00000078399.19	protein_coding	"HOXA9"	chr7:27162438-27175180	9.93462479	0.72148813	-13.76963
ENSG00000124812.15	protein_coding	"CRISP1"	chr6:49834257-49877096	9.93462479	0.72148813	-13.76963
ENSG00000148572.16	protein_coding	"NRBF2"	chr10:63133247-63155031	9.93462479	0.72148813	-13.76963
ENSG00000197728.11	protein_coding	"RPS26"	chr12:56041351-56044697	9.93462479	0.72148813	-13.76963
ENSG00000215174.2	protein_coding	"NLRP2B"	chrX:57677067-57680260	9.93462479	0.72148813	-13.76963
ENSG00000091073.19	protein_coding	"DTX2"	chr7:76461676-76505995	19.8692496	1.44297627	-13.76963
ENSG00000168484.12	protein_coding	"SFTPC"	chr8:22156913-22164479	20.5315579	1.44297627	-14.228618
ENSG00000276747.1	protein_coding	"PADI6"	chr1:17372196-17401699	20.5315579	1.44297627	-14.228618
ENSG00000143036.17	protein_coding	"SLC44A3"	chr1:94820342-94895246	21.1938662	1.44297627	-14.687606
ENSG00000100167.20	protein_coding	"SEPTIN3"	chr22:41969475-41998221	10.5969331	0.72148813	-14.687606
ENSG00000114349.10	protein_coding	"GNAT1"	chr3:50191610-50197696	10.5969331	0.72148813	-14.687606
ENSG00000129749.3	protein_coding	"CHRNA10"	chr11:3665587-3671384	10.5969331	0.72148813	-14.687606
ENSG00000136933.17	protein_coding	"RABEPK"	chr9:125200542-125234161	10.5969331	0.72148813	-14.687606
ENSG00000179041.4	protein_coding	"RRS1"	chr8:66429014-66430733	10.5969331	0.72148813	-14.687606
ENSG00000186493.13	protein_coding	"C5orf38"	chr5:2752131-2755397	10.5969331	0.72148813	-14.687606
ENSG00000267534.4	protein_coding	"S1PR2"	chr19:10221433-10231331	10.5969331	0.72148813	-14.687606
ENSG00000005073.6	protein_coding	"HOXA11"	chr7:27181157-27185232	11.2592414	0.72148813	-15.605581
ENSG00000100216.6	protein_coding	"TOMM22"	chr22:38681957-38685421	11.2592414	0.72148813	-15.605581
ENSG00000113088.6	protein_coding	"GZMK"	chr5:55024256-55034570	11.2592414	0.72148813	-15.605581
ENSG00000121716.20	protein_coding	"PILRB"	chr7:100352176-100367733	11.2592414	0.72148813	-15.605581
ENSG00000126251.6	protein_coding	"GPR42"	chr19:35370929-35372962	11.2592414	0.72148813	-15.605581
ENSG00000178093.14	protein_coding	"TSSK6"	chr19:19512418-19515548	11.2592414	0.72148813	-15.605581
ENSG00000234186.3	protein_coding	"C16orf82"	chr16:27066707-27069165	11.2592414	0.72148813	-15.605581
ENSG00000135374.11	protein_coding	"ELF5"	chr11:34478791-34525193	23.1807912	1.44297627	-16.064569
ENSG00000105141.6	protein_coding	"CASP14"	chr19:15049480-15058293	11.9215498	0.72148813	-16.523556
ENSG00000123349.14	protein_coding	"PFDN5"	chr12:53295291-53299450	11.9215498	0.72148813	-16.523556
ENSG00000159714.12	protein_coding	"ZDHHC1"	chr16:67394152-67416833	11.9215498	0.72148813	-16.523556
ENSG00000170962.13	protein_coding	"PDGFD"	chr11:103907189-104164379	11.9215498	0.72148813	-16.523556
ENSG00000186440.2	protein_coding	"OR6P1"	chr1:158560606-158570580	11.9215498	0.72148813	-16.523556
ENSG00000137225.13	protein_coding	"CAPN11"	chr6:44158811-44184401	12.5838581	0.72148813	-17.441532
ENSG00000170439.7	protein_coding	"METTL7B"	chr12:55681678-55684611	12.5838581	0.72148813	-17.441532
ENSG00000175376.9	protein_coding	"EIF1AD"	chr11:65996545-66002176	12.5838581	0.72148813	-17.441532
ENSG00000213658.12	protein_coding	"LAT"	chr16:28984826-28990784	12.5838581	0.72148813	-17.441532
ENSG00000070193.5	protein_coding	"FGF10"	chr5:44300247-44389706	13.2461664	0.72148813	-18.359507
ENSG00000165568.18	protein_coding	"AKR1E2"	chr10:4786629-4848062	13.2461664	0.72148813	-18.359507
ENSG00000168412.6	protein_coding	"MTNR1A"	chr4:186533655-186555567	13.2461664	0.72148813	-18.359507
ENSG00000179292.5	protein_coding	"TMEM151A"	chr11:66291894-66296664	13.2461664	0.72148813	-18.359507
ENSG00000182324.7	protein_coding	"KCNJ14"	chr19:48455574-48466980	13.2461664	0.72148813	-18.359507
ENSG00000097046.13	protein_coding	"CDC7"	chr1:91500851-91525764	13.9084747	0.72148813	-19.277482
ENSG00000144043.12	protein_coding	"TEX261"	chr2:70985942-70994873	13.9084747	0.72148813	-19.277482
ENSG00000167749.11	protein_coding	"KLK4"	chr19:50906352-50910738	13.9084747	0.72148813	-19.277482
ENSG00000174740.8	protein_coding	"PABPC5"	chrX:91434595-91438584	14.570783	0.72148813	-20.195458
ENSG00000132671.6	protein_coding	"SSTR4"	chr20:23035312-23039237	29.8038744	1.44297627	-20.654445
ENSG00000103707.10	protein_coding	"MTFMT"	chr15:65001512-65029639	15.2330913	0.72148813	-21.113433
ENSG00000163840.10	protein_coding	"DTX3L"	chr3:122564338-122575203	15.2330913	0.72148813	-21.113433
ENSG00000111732.11	protein_coding	"AICDA"	chr12:8602170-8612867	15.8953997	0.72148813	-22.031408
ENSG00000106635.8	protein_coding	"BCL7B"	chr7:73536356-73557690	16.557708	0.72148813	-22.949384
ENSG00000166927.13	protein_coding	"MS4A7"	chr11:60378485-60395951	17.2200163	0.72148813	-23.867359
ENSG00000077312.9	protein_coding	"SNRPA"	chr19:40750637-40765389	17.8823246	0.72148813	-24.785334
ENSG00000135925.9	protein_coding	"WNT10A"	chr2:218880852-218899581	17.8823246	0.72148813	-24.785334
ENSG00000146700.9	protein_coding	"SSC4D"	chr7:76389334-76409697	17.8823246	0.72148813	-24.785334
ENSG00000179218.14	protein_coding	"CALR"	chr19:12938578-12944489	17.8823246	0.72148813	-24.785334
ENSG00000166295.9	protein_coding	"ANAPC16"	chr10:72216000-72235860	19.2069413	0.72148813	-26.621285
ENSG00000100284.21	protein_coding	"TOM1"	chr22:35299275-35347992	20.5315579	0.72148813	-28.457236
ENSG00000149922.11	protein_coding	"TBX6"	chr16:30085793-30091924	20.5315579	0.72148813	-28.457236
ENSG00000182359.15	protein_coding	"KBTBD3"	chr11:106051098-106077459	21.8561745	0.72148813	-30.293186
ENSG00000122121.11	protein_coding	"XPNPEP2"	chrX:129738974-129769536	22.5184829	0.72148813	-31.211162
